# The Chinese herb-derived Sparstolonin B suppresses HIV-1 transcription

**DOI:** 10.1186/s12985-015-0339-8

**Published:** 2015-07-25

**Authors:** Xin Deng, Yaping Zhang, Feng Jiang, Ran Chen, Peichun Peng, Bin Wen, Jian Liang

**Affiliations:** Department of Infectious Diseases, Ruikang Hospital Affiliated to Guangxi University of Chinese Medicine, 10 Huadong Road, 530011 Nanning, Guangxi Province China

**Keywords:** Sparstolonin B, HIV transcription, TAR region

## Abstract

**Background:**

The Chines herb derived Sparstolonin B, (SsnB), is a recently identified natural compound that selectively blocks TLR2- and TLR4-mediated inflammatory signaling. But it is unknown whether this compound has any effect on HIV infection.

**Findings:**

We found that SsnB treatment blocked HIV-1 transcription via a novel mechanism that requires the TAR region. Treatment of human T cell lines or peripheral blood mononuclear cells with SsnB at 1 μM significantly inhibited HIV production. Lastly, SsnB was able to inhibit HIV in synergy with AZT.

**Conclusions:**

These data suggest that SsnB is a novel natural compound that inhibits HIV-1 transcription and may be a new drug in the treatment of HIV infection.

## Findings

Despite the success of highly active antiretroviral therapy (HAART) in containing human immunodeficiency virus (HIV) infection, there has been an urgent demand for cheaper and alternative drugs in developing countries. Moreover, HIV persists in stable reservoirs harboring chromosomally integrated latent HIV-1 proviruses, where continuous viral production and reactivation of transcription from these reservoirs are not affected by current drugs [1–4]. As such, novel classes of antivirals are needed to inhibit these processes. In this regard, a drug that blocks HIV transcription would be of great value because it offers the potential to shut down the transcription in HIV latent reservoirs.

**Fig. 1:**
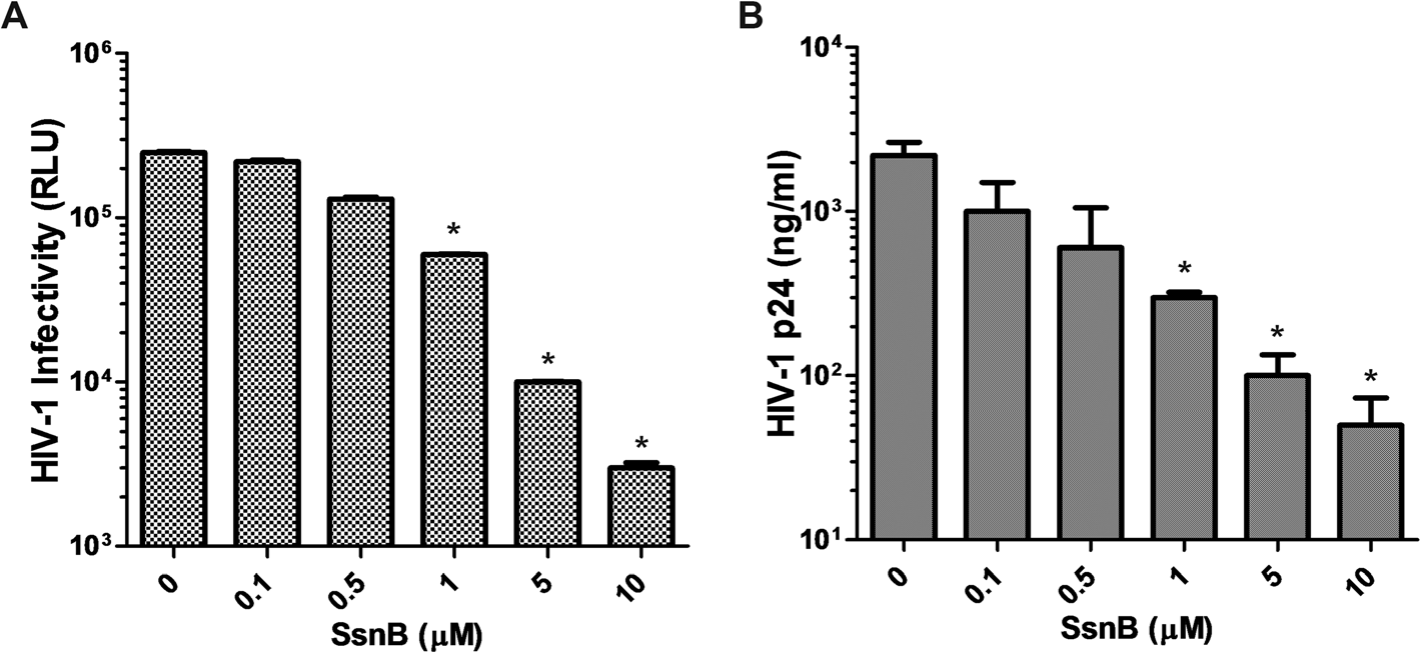
SsnB inhibits HIV production. **a** CEM-SS cells were infected with HIV pNL4.3 (MOI 0.01) and then treated with SsnB at indicated concentrations for 12 h. Newly released virus in the supernatants was collected 24 h after exposure to SsnB and then titered on the TZM-Bl cells. *p < 0.01, n = 4. **b** PHA activated PMBCs were infected with pNL4.3 (MOI 0.1) for 5 h followed by SsnB treatment for 12 h. After an additional 24 h, the HIV p24 concentrations in the supernatants were determined by ELISA. *p < 0.01, n = 3

SsnB was isolated from a Chinese herb, *Spaganium stoloniferum* [5, 6] and was recently reported to block TLR2 and TLR4 pathways [7]. Here we report that SsnB is a potent inhibitor of HIV infection. Specifically, we performed a dose response experiment by treating HIV pNL4.3 infected CEM-SS cells with various doses of SsnB. Fourty eight hours post-infection we harvested supernatants and titered the infectivity on the indicator cell line TZM-bl [8]. It was observed that SsnB treatment reduced the infectivity of the supernatants by more than 10 fold (Fig. 1a). To expand our observation, we repeated the experiment using peripheral blood mononuclear cells (PBMCs). Similarly, increasing concentrations of SsnB inhibited the production of HIV, measured by the release of p24 into the supernatants (Fig. 1b).

**Fig. 2:**
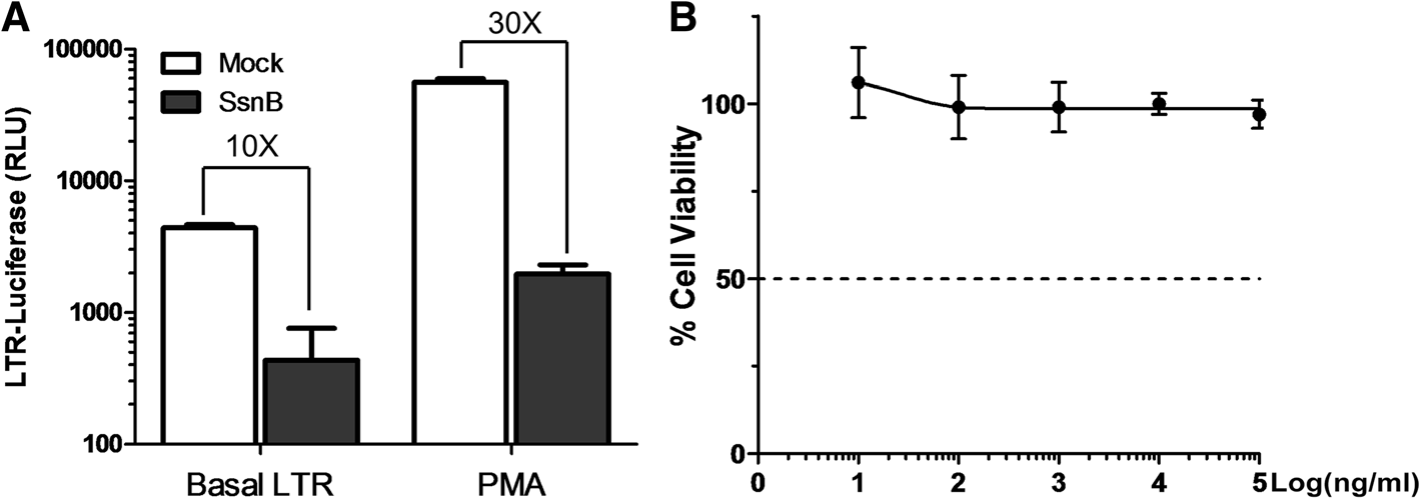
SsnB inhibited HIV LTR promoter activity. **a** 293T cells were transfected with HIV LTR plasmid together with pGL4.74 [hRluc/TK]. Cells were either left unstimulated or stimulated with PMA (50 ng/ml) for 12 h followed by mock or SsnB (1 μg/ml, ~3.7 μM) treatment for another 12 h. Luciferase assay was performed. Normalized HIV LTR promoter activity was presented. **b** CEM-SS cells were treated with SsnB at various concentrations for 12 h and cell viability was determined by CellTiter Glo kit (Promega) 24 h after the initial exposure

Next, we sought to determine if SsnB inhibits HIV-1 transcription. To this end, we performed luciferase reporter assays. 293T cells were transfected by HIV-1 LTR-driven luciferase reporter constructs and then treated by SsnB at various concentrations. HEK293T cells in 24-well plates were transfected with 0.2 μg reporter plasmid. 0.05 μg pGL4.74[hRluc/TK] was included to control for transfection efficiency. Dual luciferase assay was performed. In support of our findings, SsnB treatment indeed inhibited the luciferase activity (Fig. 2a). Of note, HIV-1 LTR transcriptional activity was significantly increased upon phorbol myristate acetate (PMA) stimulation, whereas SsnB treatment reduced it by nearly 30 fold (Fig. 2a). To ensure that the observed effect was not due to the cytotoxicity of SsnB, we sought to determine the CC_50_ of the compound and found no cytotoxicity even at the highest concentration that was tested (Fig. 2b).

**Fig. 3:**
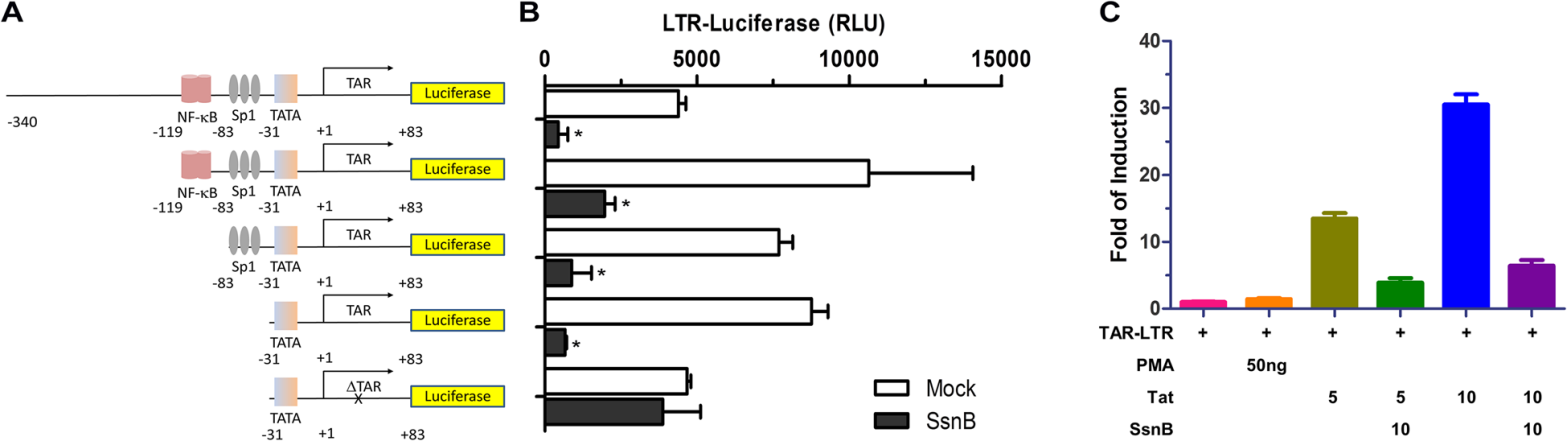
SsnB inhibition of HIV requires TAR region. **a** Illustration of successive deletion constructs that were used in this experiment. The TAR-deleted LTR was created by restriction digest to remove nucleotides downstream of +24 relative to the transcription start site. **b** 0.1 μg of the reporter constructs in (**a**) were transfected into 293T cells in the presence or absence of SsnB (1 μg/ml) for 12 h. Luciferase assays were done 48 h after transfection. *p < 0.01, n = 3. **c** 0.1 μg of the minimal TAR-LTR (−31 to +83) construct was transfected into 293T cells alone or with 5 or 10 ng Tat expressing plasmid. Twenty four hours post-transfection, two samples were treated with SsnB (1 μg/ml) for 12 h. Luciferase assay was done 24 h thereafter. Notably, this construct does not respond to PMA treatment (50 ng/ml)

To investigate the potential mechanism of inhibition, we generated successive deletion constructs by removing the two NF-B binding sites, three Sp1 binding sites, and the TAR region from HIV-1 LTR [9, 10] (Fig. 3a). All of these LTR fragments were synthesized at WuXi AppTec (China) and subcloned into the pGL4.11[luc2P] plasmid (Promega). We transfected 293T cells with these constructs and treated cells with SsnB. While the removal of the NF-κB or Sp1 binding sites has no effect on SsnB-mediated inhibition, the ΔTAR construct became non-responsive to SsnB treatment (Fig. 3b). To corroborate this finding, we co-transfected a minimal LTR-Luc construct containing the TAR region with a Tat expressing plasmid into 293T cells. In the presence of SsnB, Tat-induced LTR transcription was severely inhibited (Fig. 3c). Altogether, our results suggest that SsnB is inhibiting HIV transcription via a novel mechanism that requires the presence of TAR region. Of note, the ΔTAR construct remained transcriptionally active, albeit at much lower efficiency, which is consistent with what has been reported recently [11]. The TAR region is very important for HIV Tat-dependent transcriptional activation [12–14]. It would be interesting to test in the future whether SsnB exerts its inhibitory effect by directly interacting with TAR region or with TAR-region binding proteins such as HIV Tat or its cofactor. Further effort to identify cellular or viral targets of SsnB would be crucial in understanding the mechanism of SsnB-mediated blockage of HIV transcription.

Lastly, we tested whether SsnB is able to act synergistically with approved antivirals. To this end, we tested the IC_50_ of SsnB with the chain terminator of HIV replication, azidothymidine (AZT) [15]. Data from the drug combination experiments were analyzed according to theorem of Chou-Talalay [16, 17]. In each experiment, the dose-effect data for each single agent and the combination were used to plot a median-effect curve [16, 17]. The derived curves are used for calculation of the combination index (CI) (Table 1) as described previously [18]. The resulting combination index (CI) offers quantitative definition for additive effect (CI = 1), synergism (CI < 1), and antagonism (CI > 1) in drug combinations [16, 17]. Shown in Table 1, SsnB displayed synergy with AZT when administered together. Altogether, our findings show that SsnB is a novel natural compound that exerts anti-HIV activity by suppressing HIV transcription through the TAR region. These exciting results warrant future studies in testing its effect in combination with other known HIV drugs. It is possible that SsnB may become a new class of anti-HIV drugs that is more affordable.

**Table 1:** Synergism between SsnB and AZT

Expt no.	Concentration of:		CI at HIV-1 inhibition of:			
	AZT (μM)	SsnB (μg/ml)	50 %	75 %	90 %	95 %
1	0.04, 0.16, 0.64, 1.28	0.1, 0.5, 1, 10	0.87	0.63	0.47	0.38
2	0.0025, 0.005, 0.01, 0.02	0.5, 1, 10, 50	0.77	0.53	0.46	0.43

0.5 × 10^6^/ml CEM-SS cells were exposed to pNL4.3 virus (p24 ~ 100 ng) as inoculum in flasks containing either single agent (four concentrations each) or four combinations of AZT and SsnB (for example, in Exp 1, 0.04 μM AZT + 0.1 μg/ml SsnB; 0.16 μM AZT+ 0.5 μg/ml SsnB, and so on) for 12 h. Fourty eight hours thereafter, the production of infectious virus in the supernatants was determined by the standard TZM-bl assay [8]. The calculation was done using the method described in [18, 19]. CIs of <1, 1, and >1 indicate synergism, additive effects, and antagonism, respectively.
